# Impact of an interactive CTCA/FFR_CT_ interventional stent planning tool on patient management

**DOI:** 10.1007/s10554-025-03464-0

**Published:** 2025-07-19

**Authors:** David Murphy, Anil Gurung, Daniel McKenzie, Robert Lowe, Sri Raveen Kandan, Thomas Burchell, Kevin Carson, Ali Khavandi, Jonathan C. L. Rodrigues, JCL Rodrigues

**Affiliations:** 1 Cardiology Department, Royal United Hospitals Bath NHS Foundation Trust, Bath, UK; 2 Department for Health, University of Bath, Bath, UK; 3 Radiology Department, Royal United Hospitals Bath NHS Foundation Trust, Bath, UK; 4 Department of Radiology, Royal United Hospitals Bath NHS Foundation Trust, Combe Park, Avon, Bath, BA1 3NG UK

**Keywords:** Fractional flow reserve, Virtual stent planning, Chronic coronary syndrome

## Abstract

Fractional flow reserve (FFR) post percutaneous coronary intervention (PCI) is an important determinant of patient outcomes. A new virtual stent planning tool allows users to model PCI and obtain post PCI FFR_CT_. We hypothesised that where a sub-optimal post PCI FFR_CT_ was predicted theoretical management may be altered prior to invasive angiography. Single-centre retrospective review of patients listed for PCI with preceding CTCA(+ FFR_CT_), (Jan 2023-Sep 2024). Interventional cardiologists (ICs) at our institution determined a theoretical management strategy of (1) medical therapy (GDMT) (2) PCI or (3) multi-disciplinary team meeting (MDT) ± coronary artery bypass grafting (CABG) on a per-patient and per-vessel basis. The virtual stent planning tool was then unblinded and decision making repeated. Changes to management were compared with Wilcoxon-signed rank test and inter-rater agreement with Fleiss’ free marginal kappa. 335 patients had a CTCA(+ FFR_CT_), 96 clinically listed for angiography, 74 included in the study. 73% male, 66 ± 11 years. On a per-patient basis using CTCA(+ FFR_CT_) data PCI was chosen in 95% of cases, GDMT in 4% and MDT ± CABG in 1%. The addition of the virtual planning tool changed management strategy to PCI in 59% of cases, GDMT in 30% and MDT in 11%, Wilcoxon-signed rank test *P* = 0.04. In patients scheduled to undergo clinically indicated PCI with preceding CTCA(+ FFR_CT_) the use of the virtual stent planning tool significantly altered ICs theoretical management strategy upstream of invasive angiography, with a reduction in PCI, predominantly in favour of GDMT.

## Introduction

Angina is associated with significant morbidity and impaired health related quality of life [1, 2]. In addition to lifestyle modifications, preventive therapies, and symptom directed medical treatment, percutaneous coronary intervention (PCI) is frequently performed to alleviate symptoms [3, 4].

Computed tomography coronary angiography (CTCA) derived fractional flow reserve (FFR_CT_) has been shown to influence physician decision-making regarding both the need for further downstream ischaemia testing and the appropriateness of PCI [5, 6]. Patients with coronary artery disease (CAD) producing a modelled FFR_CT_ <0.8 are often triaged for PCI, whereas lesions with FFR_CT_ >0.8 are generally considered non-flow limiting and are managed conservatively [7].

Although a lesion may be obstructive, FFR_CT_ modelling does not predict whether PCI will normalize the pressure gradient– specifically, whether it will result in a post-PCI FFR > 0.8. Post-PCI FFR is an important determinant of clinical outcomes, including cardiac death, target vessel failure (TVF) and target vessel myocardial infarction (TVMI) [8, 9].

In the presence of serial lesions, invasive FFR measurements present additional challenges. An FFR measured distal to multiple lesions reflects the cumulative pressure drop across the vessel, rather than the impact of an individual lesion [10]. While measuring FFR at various points along the coronary artery can help estimate the relative contribution of each lesion to the pressure drop, this does not necessarily correlate with the extent of myocardial ischaemia in the perfused territory. Moreover, without the ability to model the effect of stenting, it remains uncertain whether intervention on one or multiple lesions is necessary.

From a procedural standpoint, distal lesions are often treated first to avoid crossing and potentially damaging newly stented proximal lesions. Accurate identification of which lesions require PCI would therefore be clinically valuable. Importantly, treating multiple lesions or diffuse disease typically requires longer stent lengths, which increases the risk of stent thrombosis and the need for repeat revascularization [11].

A virtual stent planning platform (HeartFlow, Redwood City, California) has recently become available, enabling users to model the placement of a virtual stent and recalculate the predicted post-procedure FFR_CT_ [12]. The accuracy of this tool has been validated against invasive FFR measurements [13]. We hypothesised that the use of the virtual stent planner tool would allow clinicians to identify cases in which a theoretical stenting would fail to achieve a satisfactory post-PCI FFR_CT_ (i.e. >0.8), or would require substantially greater stent lengths to do so. We further hypothesised that such insights could influence clinical decision-making, and we aimed to quantify any resulting changes in management decisions prompted by this technology. We additionally aimed to establish whether the planner tool could assist in catheterisation laboratory planning by predicting procedural duration, as well as the number and length of stents required.

## Methods

### Ethics

This study involved a retrospective review of clinically indicated diagnostics. It focused on theoretical management decision making only and did not impact on the patient’s actual management plan. All patients were consented as normal for their invasive angiogram. As per the NHS Research Authority decision tool [14] no written informed consent was obtained, and no ethical committee approval was deemed necessary. The study was registered as a service evaluation with the trusts audit department waiving the need for formal written consent. Patients and public were not involved in the design, conduct, reporting or dissemination of our research. No funding has been received for this work.

### Setting

Our centre is a medium sized hospital in the United Kingdom healthcare system, with a bed capacity of approximately 760 and a catchment population of 500,000 people. The cardiac centre sees approximately 250 patients per month through its rapid access chest pain clinic (RACPC) and undertakes approximately 450 PCI procedures per year including primary PCI and chronic total occlusion (CTO) procedures.

### CTCA acquisition

All CTCA imaging was done as part of standard of care. Oral beta blockers or ivabradine were used targeting a heart rate < 60 beats per minute with additional intravenous beta blockers used as necessary. Unless any contraindications 800 µg sub-lingual nitrate was used as a vasodilator. CTCA Studies were acquired with a 128-slice CT scanner (Siemens SOMATOM Drive, Siemens Healthineers, Erlangen). Imaging protocol involved a test bolus technique (12 ml Niopam 340 at 6 ml/sec) then full acquisition (60-80 ml Niopam 340 at 6 ml/sec). A slice thickness of 0.6 mm, a pitch sequential scan feed of 34.5 mm, rotation time 0.28s, and a tube voltage reference, depending on body habitus, of 100kVp with automated kV modulation and 220 reference mAs with automated tube current modulation. Default CTCA reconstructions were cardiac field of view 0.6 mm coronary vascular reconstruction kernel (Bv38, Siemens Healthineers), 0.6 mm sharp vascular reconstruction kernel (Bv45, Siemens Healthineers) and 0.6 mm raw axial reconstructions without automated smoothing between steps (Truestack I30f, Siemens Healthineers). All CTCAs were reported clinically as per Coronary Artery Disease Reporting and Data Systems 2.0 (CAD-RADS) using syngo.via post-processing software (Siemens Healthineers, Erlangen) by consultant cardiothoracic radiologists with at least 10 years’ CTCA experience. FFR_CT_ was requested at the discretion of the reporting radiologist. This additional testing is provided externally by HeartFlow Inc. as previously described [12, 15].

### Management strategies

A prospectively maintained database consisting of patients referred for a clinically indicated ICA with a preceding CTCA and FFR_CT_ was retrospectively reviewed (Jan 2023-Sep 2024). The decision to list for an ICA was at the discretion of the treating cardiologist and this study did not interfere with this pathway.

Interventional cardiologists (ICs) were asked to independently determine a theoretical management plan for each patient. Demographic data, presenting complaint, cardiovascular risk factors and left ventricular function was made available to them. They were additionally given the CTCA and FFR_CT_ report together with the corresponding interactive FFR_CT_ images but not stent planner. Management decisions were divided into (1) guideline directed medical therapy [GDMT]); (2) PCI (in addition to GDMT); (3) multi-disciplinary meeting (MDT) with a view to possible coronary artery bypass graft (CABG) surgery. Decision making was sought on a per-vessel and per-patient basis. One management option had to be chosen.

The interactive virtual stent planning tool was then immediately unblinded and the IC were able to interact with it. The planning software allows user to place a theoretical stent(s) of any length, at any point along any coronary artery. The software then modifies the anatomic lumen boundary (based on the CTCA imaging) to simulate no stenosis and recalculates a theoretical post PCI FFR_CT_ in a live fashion. The ICs were allowed to plan any theoretical PCI scenario they wanted. They were then asked to repeat the management decisions on a per-patient and per-vessel basis, as above.

**Fig. 1:**
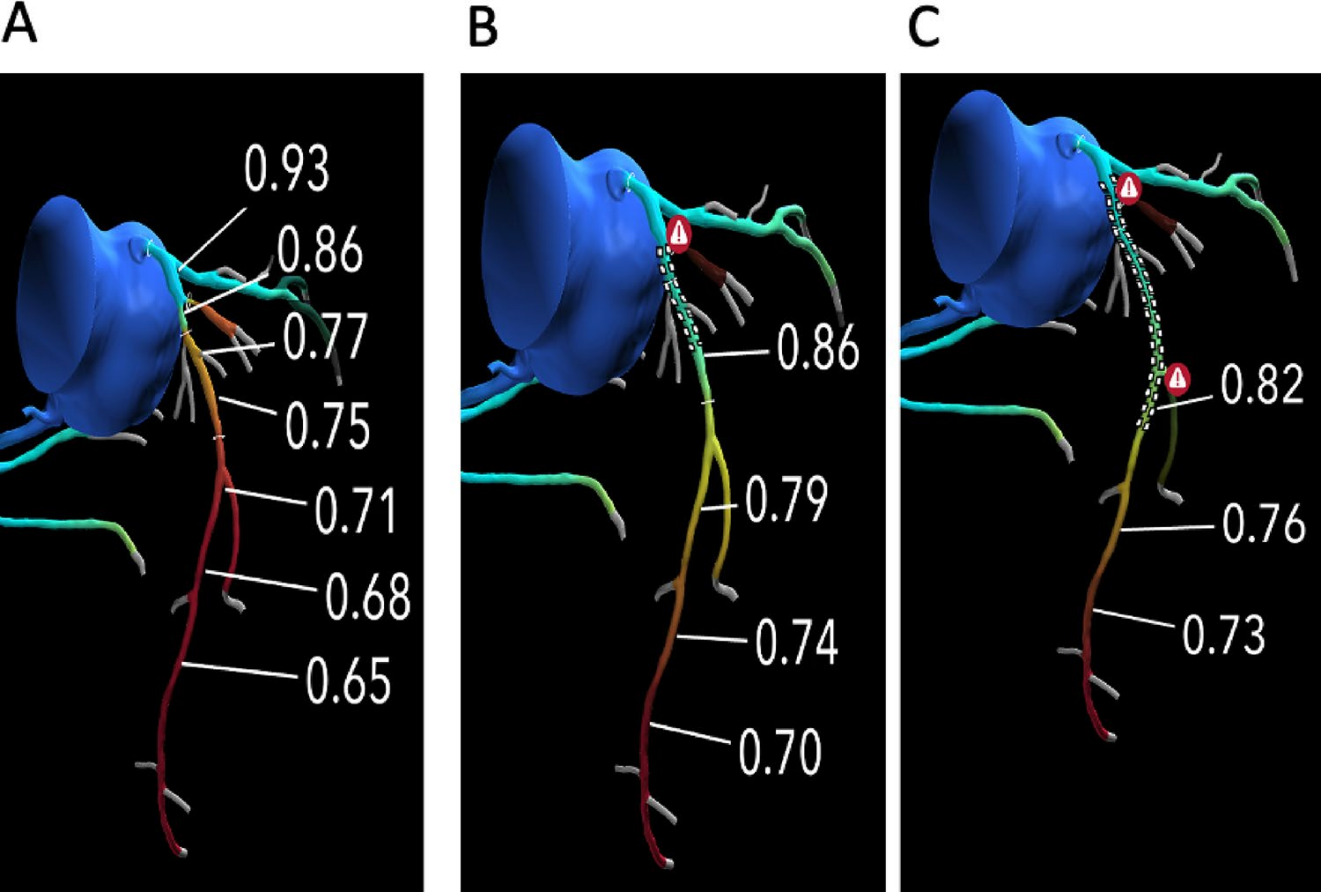
(**A**) CTCA with an anatomical severe stenosis and subsequent moderate stenosis in the LAD. (**B**) Modelled PCI with post PCI FFR_CT_ improving to 0.86 across the proximal lesion but FFR_CT_ <0.8 distally. (**C**) Modelled extension of the stented segment across both lesions with a post PCI-FFR_CT_ remaining sub-optimal. Indicative of diffuse coronary artery disease unlikely to benefit from PCI. Abbreviations: CTCA = computed tomography coronary angiogram.FFR_CT_=CT based fractional flow reserve.PCI = percutaneous coronary intervention. LAD = left anterior descending

Decision making was sought from a pool of 6 interventional cardiologists (each with > 5 years of consultant experience). On multiple occasions during the study period a maximum of five patients from the database were analysed at any one time. A random selection of three ICs was made on each occasion, thus increasing the variety of ICs undertaking the decision making. The final theoretical management decision recorded was based on the majority decision amongst the three. Cases where the virtual planner tool was not possible in any of the major epicardial vessels were excluded as were cases where a total occlusion was modelled at CTCA. Vessels of interest were the left main stem (LMS), left anterior descending (LAD), left circumflex (LCx) and right coronary arteries (RCA). A representative example is given in Fig. 1.

### Catheterisation laboratory logistics

In cases where PCI was chosen, following the use of the planner tool, the reviewers were additionally asked to specify the number and length(s) of stent(s) together with an estimate of how long such a procedure would take in the catheterisation laboratory. Timings were categorised as < 60 min, 60–120 min and > 120 min. For cases then underwent ICA during the study period a comparison with real-world outcomes has been reported.

### Statistics

Statistical calculations were carried out using SPSS (IBM Corp. IBM SPSS Statistics for Mac, Version 29.0.2.0, Armonk, NY: IBM Corp). A Shapiro-Wilks test was used to assess for normality of the data. Continuous variables are presented with mean and standard deviation (SD) and those with extreme outliers with median and interquartile range (IQR). Fleiss’ free kappa was used to determine inter-rater agreement with regard to theoretical management decisions. A two-tailed Wilcoxon-signed rank test was used to establish if there was statistical difference between overall management decisions. Chi-square testing and t-testing were used to compare PCI procedural duration, stent number and length. Significance was determined based on a 2-sided P value < 0.05.

## Results

### Patient population

**Fig. 2:**
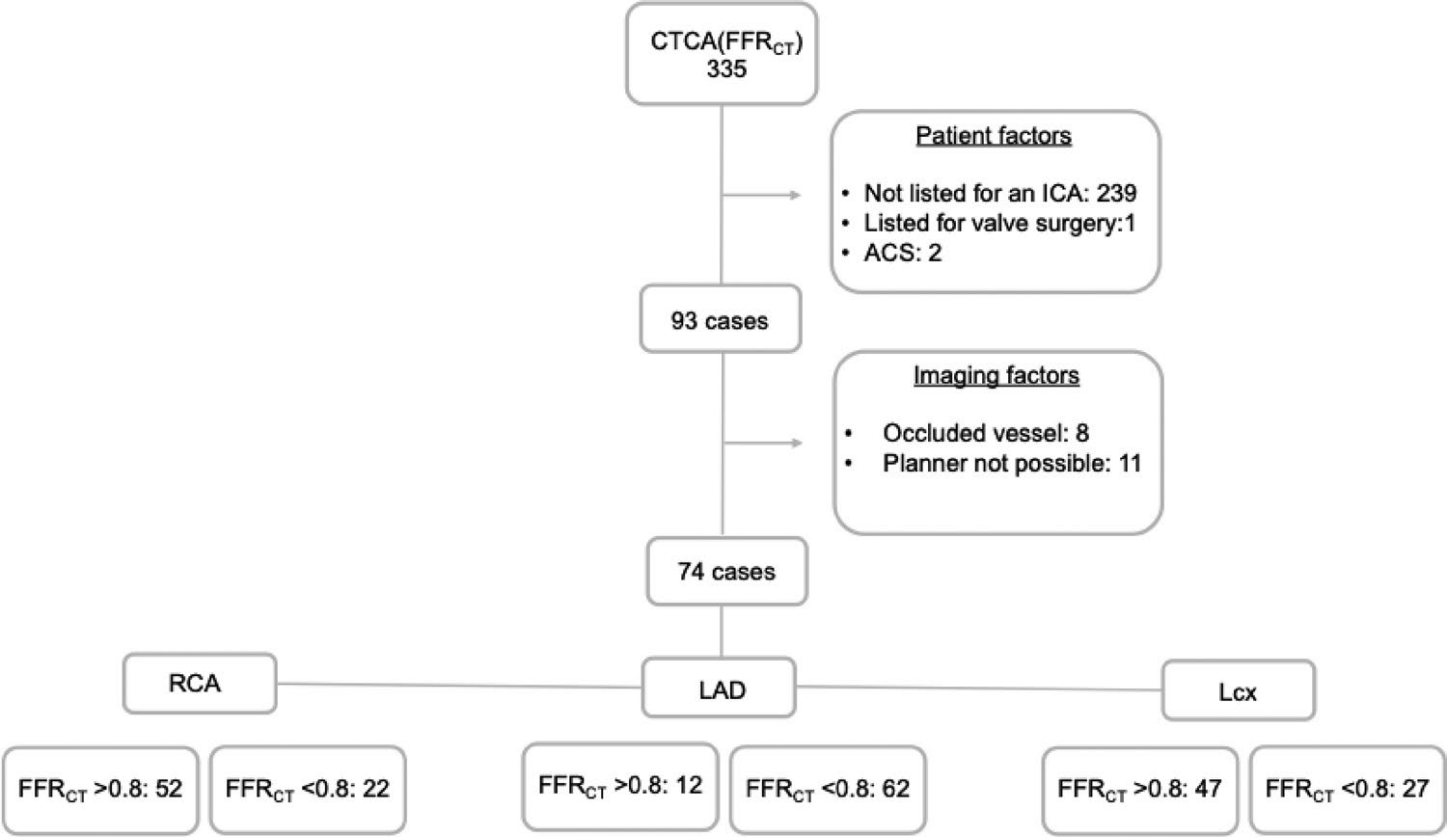
Study flow chart. Abbreviations: CTCA = Computed Tomography Coronary Angiogram.FFR_CT_=CT based fractional flow reserve.ACS = acute coronary syndrome

74 patients were included in the study, 73% (54/74) male, 66 ± 11 years. A study flow chart is presented in Fig. 2 together with details of the study population in Table 1.

**Table 1:** Patient demographics and CTCA imaging results on a per-case basis. Management subdivisions are based on the those chosen using the PCI planning tool. Smoking history: current or former

Demographics	Total *n*=74	GDMT *n*=22	PCI *n*=44	MDT ±CABG *n*=8
Male, n (%)	54 (73)	15 (68)	31 (70)	8 (100)
Years of age, mean (±SD)	66 (±11)	69 (±9)	65 (±12)	66 (±12)
Diabetes mellitus, n (%)	13 (18)	1 (5)	11 (25)	1 (13)
Hypertension, n (%)	44 (59)	16 (73)	24 (55)	4 (50)
Family history of premature CAD, n (%)	21 (28)	5 (23)	14 (32)	2 (25)
Smoking history, * n (%)	35 (47)	14 (64)	16 (36)	5 (63)
Dyslipidaemia, n (%)	43 (58)	16 (73)	22 (50)	5 (63)
Body mass index in kg/m^2^, median (±IQR)	28 (±4.1)	27 (±4.3)	28 (±4.6)	27 (±4.4)
Presentation, n (%) Typical angina Atypical angina Non-angina	44 (59) 28 (38) 2 (3)	10 (45) 12 (55) -	28 (64) 14 (32) 2 (4)	6 (75) 2 (25)
Left ventricular ejection fraction, median (±IQR)	55 (±5)	55 (±3)	57 (±5)	55 (±7)
CTCA per case				
CAD-RADS 3, n (%)	12 (17)	8 (36)	5 (11)	-
CAD-RADS 4a, n (%)	53 (76)	14 (64)	37 (84)	5 (63)
CAD-RADS 4b, n (%)	5 (7)	-	2 (5)	3 (37)

Abbreviations: GDMT = guideline directed medical therapy, PCI = percutaneous coronary intervention, MDT = multidisciplinary team meeting, CABG = coronary artery bypass graft surgery, CTCA = computed tomography coronary angiogram, CAD-RADS = coronary artery disease reporting and data system, SD = standard deviation, IQR = interquartile range, CAD = coronary artery disease

### CTCA results

Tables 1 and 2 detail the CTCA findings for the study population on a per-vessel and per-patient basis. A reported CAD-RADS grade of 4b defined three vessel coronary artery disease and/or significant left main stenosis.

**Table 2:** CTCA findings on a per-vessel basis

Group	Vessel	Lesion specific FFR_CT,_ (±SD)	End vessel FFR_CT,_ (±SD)	Segment	≤Mild stenosis, *n* (%)	Moderate stenosis, *n* (%)	Severe stenosis, *n* (%)
GDMT *n*=22	LAD, n (%)	0.75 ±0.8	0.7 ±0.9	Proximal Mid Distal	11 (50) 3 (36) 16 (73)	10 (45) 5 (23) 5 (23)	1 (5) 9 (41) 1 (5)
	Lcx, n (%)	0.86 ±0.07	0.84 ±0.08	Proximal Mid Distal	19 (86) 20 (90) 21 (95)	2 (9) 1 (5) 1 (5)	1 (5) 1 (5) -
	RCA, n (%)	0.86 ±0.1	0.86 ±0.09	Proximal Mid Distal	20 (91) 18 (82)	2 (9) 3 (14)	- 1 (5)
PCI *n*=44	LAD, n (%)	0.69 ±0.12	0.65 ±0.11	Proximal Mid Distal	17 (39) 21 (48) 37 (84)	13 (30) 7 (16) 5 (11)	14 (32) 6 (36) 2 (5)
	Lcx, n (%)	0.82 ±0.14	0.8 ±0.14	Proximal Mid Distal	35 (80) 31 (70) 37 (84)	5 (11) 7 (16) 3 (7)	4 (9) 6 (14) 4 (9)
	RCA, n (%)	0.83 ±0.15	0.81 ±0.15	Proximal Mid Distal	36 (82) 32 (73) 39 (89)	3 (7) 6 (14) 3 (7)	5 (11) 6 (14) 2 (5)
MDT± CABG *n*=8	LAD, n (%)	0.62 ±0.1	0.59 ±0.08	Proximal Mid Distal	- 4 (50) 7 (88)	3 (38) 1 (13) 1 (13)	5 (63) 3 (38) -
	Lcx, n (%)	0.73 ±0.2	0.69 ±0.18	Proximal Mid Distal	3 (38) 4 (50) 6 (75)	4 (50) 3 (38) 1 (13)	1 (13) 1 (13) 1 (13)
	RCA, n (%)	0.77 ±0.14	0.76 ±0.14	Proximal Mid Distal	5 (63) 5 (63) 4 (50)	- 1 (13) -	3 (38) 2 (25) 4 (50)

Abbreviations: LAD = left anterior descending artery, Lcx = left circumflex artery, RCA = right coronary artery, GDMT = guideline directed medical therapy, PCI = percutaneous coronary intervention, MDT = multidisciplinary team meeting, CABG = coronary artery bypass graft surgery, SD = standard deviation, IQR = interquartile range, FFR_CT_=CT based fractional flow reserve

### Patient management strategies

**Fig. 3:**
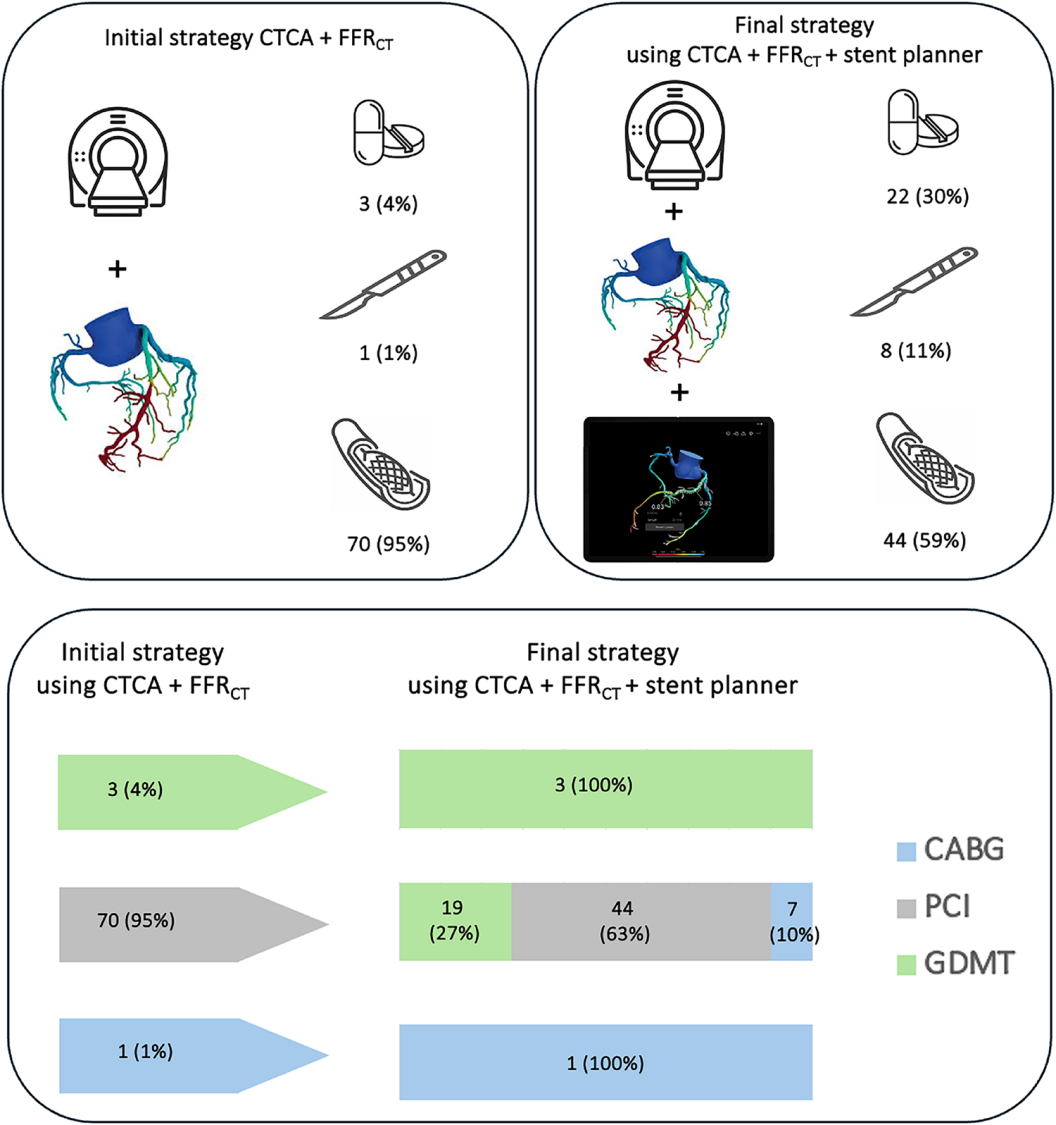
Change in theoretical decision making based on CTCA (FFR_CT_) and subsequently with the additional use of the virtual planning tool on a per-patient basis for the overall cohort (top panel) and subdivided by initial and subsequent management strategies (bottom panel)

On a per-patient basis using the CTCA and FFR_CT_ data PCI was chosen in 95% (70/74) of cases, GDMT in 4% (3/74) and MDT with a view to possible CABG in 1% (1/74). With the addition of the virtual stent planning tool management decision were changed to PCI in 59% (44/74) of cases, 30% (22/74) GDMT and 11% (8/74) MDT, Wilcoxon-signed rank test *P* = 0.04, (see Fig. 3).

The Fleiss’ free marginal kappa inter-rater correlation between ICs when using the CTCA (FFR_CT_) data was 0.75 (95% CI 0.61–0.89). IC inter-rater correlation of decision making when using the virtual stent planner was 0.64 (95% CI 0.52–0.75). This equated to an overall agreement between the three interventional cardiology raters of 83% and 76% respectively.

### Per-vessel management strategies

**Fig. 4:**
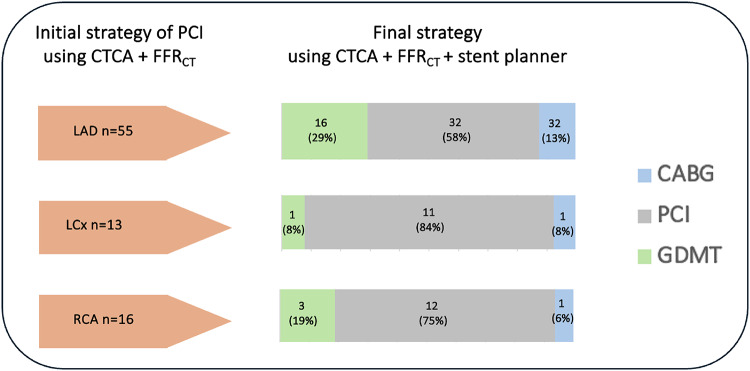
Change in theoretical decision making based on CTCA (FFR_CT_) and subsequently with the additional use of a virtual stent planner on a per-vessel basis

On a per-vessel basis an initial PCI management strategy was chosen for 84 vessels, 65% (55/84) LAD, 16% (13/84) LCx and 19% (16/84) RCA. The change in decision making following the use of the virtual stent planner is given in Fig. 4.

### Correlation with real world outcomes

During the study period 42 (57%) cases underwent invasive angiography. Following an angiogram PCI was undertaken in 55% (23/42), GDMT in 26% (11/42) and referral for MDT ± CABG in 19% (8/42).

In cases where PCI was chosen as a management strategy using the planning tool and who subsequently underwent PCI (*n* = 18) during the study period, the predicted procedural time was not statistically different from that observed when modelled on an ordinal scale (*P* = 0.15). There was no difference in the median stent number or length between that predicted and that observed in this small cohort on a per-patient basis; *P* = 0.4 and 0.4 respectively.

## Discussion

The use of a virtual stent planner significantly altered theoretical patient management with a reduction in the frequency with which PCI was offered, predominantly in favour of GDMT, mostly in the LAD.

Previous work has looked at how the virtual stent planner alters decision making but this was at the time of invasive angiography [16]. In addition to offering GDMT, PCI and CABG as decision options the published work by van Belle et al. also asked the reviewers whether an invasive FFR assessment was required, thereby including the value added by FFR_CT_ rather than the virtual planning tool alone. Our study was the first study to look specifically at the role of the virtual stent planner upstream in the patient pathway over and above the impact of CTCA and FFR_CT_ results.

The impact of PCI on symptom burden is poorly understood. Indeed, FFR is only a modest predictor of angina burden [17]. The ORBITA 2 study demonstrated the clinical efficacy of PCI in the setting of a randomised sham-controlled study [18]. In the absence of anti-anginal medical treatment a significant improvement in angina symptom burden was reported relative to a placebo. When put in the context of ORBITA 1 [19], where a negligible improvement in angina was shown when PCI was used in addition to medication, the ORBITA investigators have raised the question as to the rationale for PCI as a second line therapy. Targeting focal disease where a predicted short stent achieves an excellent post-procedure FFR_CT_ may provide some guidance as to the merits of such a strategy, even as first line. In contrast diffuse disease and/or modelled PCI that does not achieve a reasonable post-PCI FFR_CT_ may re-orientate physicians towards the merits of an aggressive medical approach.

This was a single centre study analysing the decision making of interventional cardiologists but in day-to-day practice the decision to list for an ICA is often made by non-interventional cardiologists so findings here may not be generalisable to routine practice. We acknowledge that all decisions were theoretical in nature. Our centre incorporated FFR_CT_ into the patient pathway in 2018 [6] so both the radiologists and cardiologists in the study are familiar with the technology and any pre-existing bias cannot be excluded. A large proportion of our cohort had proximal LAD disease. This remains a highly weighted lesion in the guidelines and may therefore have influenced decision making favouring a more aggressive intervention than might be expected for stenosis elsewhere even with sub-optimal post procedural FFR_CT_. Although pre-PCI measurement of FFR is guideline recommended the use of post-PCI FFR evaluation is not routine clinical practice which may have impacted on decision making. We chose to only use patients already listed for PCI for inclusion in the study to isolate the effect of the stent planning technology over and above that of the FFR_CT_, we acknowledge this could potentially introduce a bias in favour of the stent planning technology.

## Conclusion

To our knowledge, this is the first study showing that virtual stent planning can alter decisions upstream of the catheterisation laboratory, reducing planned PCI in favour of GDMT. In a small subset, stent planner predictions closely matched real-world stent number and lengths, however, further multicentre prospective studies are needed.

## Data Availability

The data supporting the findings of this study are available upon reasonable request.

## References

[1] Knuuti J, Wijns W, Saraste A, ESC Scientific Document Group (2019). ESC Guidelines for the diagnosis and management of chronic coronary syndromes. Eur Heart J. 2020. 14;41(3):407–477. 10.1093/eurheartj/ehz425 10.1093/eurheartj/ehz425 31504439

[2] Rieckmann N, Neumann K, Feger S et al (2020) Health-related qualify of life, angina type and coronary artery disease in patients with stable chest pain. Health Qual Life Outcomes. 14;18(1):140. 10.1186/s12955-020-01312-4. Erratum in: Health Qual Life Outcomes. 2020;18(1):205. https://doi.org/10.1186/s12955-020-01443-8 10.1186/s12955-020-01312-4 PMC7222590 32410687

[3] Owlia M, Dodson JA, King JB et al (2019) Angina severity, mortality, and healthcare utilization among veterans with stable angina. JAHA 8(15). 10.1161/JAHA.119.012811 10.1161/JAHA.119.012811 PMC6761668 31362569

[4] Stable angina management. Clinical guideline [CG126] Published: 23 July 2011 Last updated: 25 August 2016

[5] Fairbairn TA, Nieman K, Akasaka T et al (2018) Real-world clinical utility and impact on clinical decision-making of coronary computed tomography angiography-derived fractional flow reserve: lessons from the ADVANCE registry. Eur Heart J 39(41):3701–3711. 10.1093/eurheartj/ehy530 30165613 10.1093/eurheartj/ehy530 PMC6215963

[6] Graby J, Metters R, Kandan SR et al (2021) Real-world clinical and cost analysis of CT coronary angiography and CT coronary angiography-derived fractional flow reserve (FFR_CT_)– guided care in the National health service. Clin Rad 76(11). 10.1016/j.crad.2021.06.009 10.1016/j.crad.2021.06.009 34261595

[7] De Bruyne B, Pijls N, Kalesan B et al (2012) FAME 2 trial investigators. FAME 2 trial investigators. Fractional flow reserve-guided PCI versus medical therapy in stable coronary disease. N Engl J Med 367:991–1001. 10.1056/NEJMoa1408758 22924638 10.1056/NEJMoa1205361

[8] Hwang D, Koo BK, Zhang J et al (2022) Prognostic implications of fractional flow reserve after coronary stenting: a systematic review and meta-analysis. JAMA Netw Open 5:e2232842. 10.1001/jamanetworkopen.2022.32842 36136329 10.1001/jamanetworkopen.2022.32842 PMC9500557

[9] Rimac G, Fearon WF, De Bruyne B et al (2017) Clinical value of post–percutaneous coronary intervention fractional flow reserve value: A systematic review and meta-analysis. American Heart Journal.;183:1–9. 10.1016/j.ahj.2016.10.005 10.1016/j.ahj.2016.10.005 27979031

[10] Ilic I, Timcic S, Odanovic N, Otasevic P, Collet C (2023) Serial stenosis assessment-can we rely on invasive coronary physiology. Front Cardiovasc Med 10:1172906. 10.3389/fcvm.2023.1172906 37200979 10.3389/fcvm.2023.1172906 PMC10185833

[11] Van Hemert N, Stella PR, Rozemeijer R et al (2022) Stent length and -diameter and long-term clinical outcomes following percutaneous coronary intervention with drug-eluting stent implantation. Eur Heart J 43(2). 10.1093/eurheartj/ehac544.2041

[12] Modi BN, Sankaran S, Kim HJ et al (2019) Predicting the physiological effect of revascularization in serially diseased coronary arteries. Circ Cardiovasc Interv 12(2):e007577. 10.1161/CIRCINTERVENTIONS.118.007577 30722688 10.1161/CIRCINTERVENTIONS.118.007577 PMC6794156

[13] Sonck J, Nagumo S, Norgaard BL et al (2022) Clinical validation of a virtual planner for coronary interventions based on coronary CT angiography. JACC Cardiovasc Imaging 15(7):1242–1255. 10.1016/j.jcmg.2022.02.003 35798401 10.1016/j.jcmg.2022.02.003

[14] NHS Health Research Authority (2024) Decision tools - is my study research? Published 2020. Available at http://hra-decisiontools.org.uk/research. Accessed Jun

[15] Taylor CA, Fonte TA, Min JK (2013) Computational fluid dynamics applied to cardiac computed tomography for noninvasive quantification of fractional flow reserve: scientific basis. J Am Coll Cardiol 61:2233–2241. 10.1016/j.jacc.2012.11.083 23562923 10.1016/j.jacc.2012.11.083

[16] van Belle E, Raposo L, Bravo Baptista S et al (2021) Impact of an interactive CT/FFR_CT_ interventional planner on coronary artery disease management decision making. JACC Cardiovasc Imaging 14(5):1068–1070. 10.1016/j.jcmg.2020.09.040 33454253 10.1016/j.jcmg.2020.09.040

[17] Cook CM, Ahmad Y, Howard JP, Shun-Shin MJ, Sethi A, Clesham GJ et al (2019) Association between physiological stenosis severity and Angina-Limited exercise time in patients with stable coronary artery disease. JAMA Cardiol 4(6):569–574. 10.1001/jamacardio.2019.1139 31042268 10.1001/jamacardio.2019.1139 PMC6495364

[18] Rajkumar CA, Foley M, Ahmed-Jushuf F et al (2023) A Placebo-Controlled trial of percutaneous coronary intervention for stable angina. N Engl J Med 389(25):2319–2330. 10.1056/NEJMoa2310610 38015442 10.1056/NEJMoa2310610 PMC7615400

[19] Al-Lamee R, Thompson D, Dehbi HM et al (2018) ORBITA investigators. Percutaneous coronary intervention in stable angina (ORBITA): a double-blind, randomised controlled trial. Lancet.;391(10115):31–40. doi: 10.1016/S0140-6736(17)32714-9. Epub 2017 Nov 2. Erratum in: Lancet. 2018;391(10115):30. 10.1016/S0140-6736(17)33366-4 10.1016/S0140-6736(17)32714-9 29103656

